# Comment regarding: what is the efficacy of aerobic exercise versus strength training in the treatment of migraine? A systematic review and network meta‑analysis of clinical trials

**DOI:** 10.1186/s10194-022-01522-9

**Published:** 2022-11-18

**Authors:** Junhee Han, Soo-Jin Cho

**Affiliations:** 1 Department of Statistics, Hallym University, Gangwon, Korea; 2 Department of Neurology, Dongtan Sacred Heart Hospital, Hallym University College of Medicine, Keun Jae Bong-gil 7, Gyeonggi-do 18450 Hwaseong, Korea

**Keywords:** Exercise, meta-analysis, Migraine, Strength training, Prevention

## Abstract

In Woldeamanuel and Oliveira (2022)’s article about the efficacy of exercise in the treatment of migraine, the ranking of the efficacy of strength training (mean difference, − 3.55), aerobic exercise (mean difference, − 2.18 to − 3.13), topiramate (mean difference, − 0.98), and amitriptyline (mean difference, 3.82) using network meta-analysis can mislead readers. First, the inclusion criteria were reported at a monthly frequency of migraine and the end of the intervention, but some article did not meet the inclusion criteria or had data inconsistency. Second, there was an inconsistency in the placebos used in the included studies, which can be problematic in network meta-analysis. Third, all three articles on strength training were rated as high-risk or exhibited some risk of bias. Finally, the effectiveness of this statistical method is questionable for assessing physical activities because strength training, aerobic exercise, and preventive medications can be simultaneously recommended for possible synergistic effects in the prevention of migraine.

Dear Editor,

I congratulate Woldeamanuel and Oliveira (2022) on their article about the efficacy of exercise in the treatment of migraine [1]. The importance of exercise as a non-pharmacological preventive therapy should be emphasized in daily practice [2], and I believe this article encourages strength training as well as aerobic exercise for migraine prevention. A detailed description of study selection and network meta-analysis in the method section and information about the exercise protocol in the discussion section are very helpful and impressive. However, the ranking of the efficacy of strength training (mean difference, − 3.55), aerobic exercise (mean difference, − 2.18 to − 3.13), topiramate (mean difference, − 0.98), and amitriptyline (mean difference, 3.82) using network meta-analysis can mislead readers.

First, the inclusion criteria were reported at a monthly frequency of migraine and the end of the intervention. Only three of the requested eight corresponding authors provided migraine-specific data. Moreover, the network meta-analysis was done with 21 articles instead of 16 articles. Monthly migraine days were different from attack frequency or headache days, and we observed inconsistency between supplementary Table 1 and the original data. The data used from a study by Varkey et al. included the mean differences in attack frequency instead of monthly migraine days [3]. Similarly, the data incorporated from another report by Benatto et al. comprised the mean differences in headache frequency instead of monthly migraine days [4].

Second, the network meta-analysis compared and ranked the efficacy of the intervention based on the assumption of a common placebo. However, I noticed an inconsistency in the placebos used in the included studies, which can be problematic in network meta-analysis. For example, Benatto et al. used a sham ultrasound as a placebo, Varkey et al. used relaxation, whereas Santiago et al. did not use a placebo [3–5]. Additionally, incorporating only one article, in each arm in the network plot on the efficacy of topiramate or amitriptyline (Fig. 2; Table 1) might have weakened the power of this analysis. Network meta-analysis also has its drawbacks, and naive ranking could be misleading if based on the probability of being the best without considering the quality of the original article [6].

Third, all three articles on strength training were rated as high-risk or exhibited some risk of bias according to supplementary Fig. 2 Aslani et al. (observed mean difference − 7.32 [− 10.50 to − 4.14]) allowed the use of drugs (nortriptyline, duloxetine, propranolol, dexamethasone, gabapentin, and venlafaxine) with a sample size of only 10 participants in each group. Therefore, the promising efficacy of strength training can be biased owing to uncontrolled oral preventive medications or small sample size [7]. Sun et al. enrolled patients with vestibular migraine and evaluated vertiginous attacks instead of headache days [8]. Strength training was not prohibited during the studies regarding aerobic exercise, and vice versa. Finally, unlike clinical trials on medications, network meta-analysis of physical activity, such as strength training or aerobic exercise, may be difficult to interpret.

A clinical trial on exercise has many challenges, including enrolling and maintaining the participants, selection of a reliable placebo, and abiding by protocols of intervention; so, a systematic review like this article can be informative, and more clinical trials on the efficacy of exercise are warranted. However, until a well-designed large-scale trial of lifestyle intervention with a reasonable follow-up duration is accomplished, a meta-analysis should be performed with caution. Network meta-analysis may be a preferred statistical tool for the selection of the most appropriate treatment from a number of options. However, the effectiveness of this statistical method is questionable for assessing physical activities because strength training, aerobic exercise, and preventive medications can be simultaneously recommended for possible synergistic effects in the prevention of migraine.

## Data Availability

The data used in the present study are available from the corresponding author on reasonable request.
